# Maternal Experience Does Not Predict Fear Extinction and Anxiety-Like Behaviour in Primiparous Rats Post-weaning

**DOI:** 10.3389/fgwh.2021.742337

**Published:** 2021-12-17

**Authors:** Jodie E. Pestana, Tayla B. McCutcheon, Sylvia K. Harmon-Jones, Rick Richardson, Bronwyn M. Graham

**Affiliations:** School of Psychology, University of New South Wales (UNSW) Sydney, Sydney, NSW, Australia

**Keywords:** anxiety disorders, fear extinction, anxiety-like behaviour, estrous cycle, reproductive experience, maternal experience, pregnancy

## Abstract

Reproductive experience leads to long-lasting changes in anxiety-like behaviour and fear extinction, the laboratory model of exposure therapy for anxiety disorders. For example, fear extinction is influenced by estrous cycle in nulliparous (no reproductive experience) female rats, but this effect is abolished in primiparous (one reproductive experience) females. It is unclear whether such changes are driven by pregnancy, maternal experience of caring for offspring during the postpartum period, or a combination of both experiences. The present study sought to determine the influence of maternal experience (i.e., exposure to pups and mother-pup interactions) on fear extinction in primiparous rats. In Experiment 1, we tested whether pup exposure is necessary to mitigate estrous effects on fear extinction in primiparous rats. Age-matched nulliparous rats, primiparous rats, and primiparous rats who experienced pregnancy but not pup exposure, underwent fear conditioning on day 1 (2 months post-parturition), extinction training during proestrus (high sex hormones) or metestrus (low sex hormones) on day 2, and extinction recall on day 3. Replicating past research, nulliparous rats showed impaired extinction recall when they were extinguished during metestrus compared to proestrus. In contrast, primiparous rats with and without pup exposure showed comparable extinction recall irrespective of estrous phase. In Experiment 2, we assessed whether naturally-occurring variation in mother-pup interactions predict future fear extinction performance and anxiety-like behaviour. During the first week of lactation, primiparous rats were measured for maternal behaviours toward pups. Primiparous rats were then tested on the light-dark box and elevated plus maze to measure anxiety-like behaviour and underwent a fear extinction protocol 1 month post-weaning. We found no significant correlations between maternal behaviour and fear extinction outcomes or anxiety-like behaviour. Our findings suggest that pregnancy, not maternal experience, mitigates the impact of estrous cycle on fear extinction. In addition, natural variation in maternal experience does not appear to contribute to variability in future fear extinction outcomes or anxiety-like behaviour in primiparous rats.

## Introduction

Women are twice as likely to be diagnosed with an anxiety disorder compared to men (1), and they experience a greater symptom severity, burden of illness, and comorbidity between disorders (2). The vulnerability to develop an anxiety disorder fluctuates over the female reproductive lifespan with the greatest risk occurring during the postpartum time (3, 4). Indeed, ~85% of women in the United States of America will become a mother before the age of 44 with two thirds of these women experiencing more than one birth (5), and 15–20% of mothers report an onset of anxiety symptoms after giving birth (6, 7). Despite this, animal studies investigating the development and treatment of anxiety disorders have primarily been conducted in male rats (8), and the few studies that have included females have mostly used female rats without prior reproductive experience. This includes studies on fear extinction, which forms the laboratory basis of exposure therapy, a highly effective treatment for anxiety disorders (9).

Exposure therapy involves the gradual presentation of anxiety-provoking stimuli in absence of a negative outcome. Over repeated exposures, patients exhibit reduced anxiety as they learn that the feared stimuli are, in fact, safe. Like exposure therapy, fear extinction involves repeatedly presenting subjects with a feared conditioned stimulus (CS, a stimulus that was previously paired with an aversive outcome) without the aversive outcome (unconditioned stimulus; US), which typically results in a decrease in fear responses to the CS. Long-term recall of extinction is assessed by re-presenting the extinguished CS at a later time, where good extinction recall is indexed by low fear responses. Understanding the factors that influence fear extinction in females across different stages of their reproductive lifespan may aid in optimising exposure therapy outcomes in women.

Over the past decade, studies have demonstrated that estradiol (i.e., an oestrogen steroid hormone) facilitates fear extinction in females without prior reproductive experience. For instance, naturally cycling female rats and women show impaired extinction recall when they have undergone fear extinction during estrous/menstrual phases of low endogenous estradiol relative to estrous/menstrual phases of high endogenous estradiol (10–20). Administration of estradiol improves extinction recall in female rats and women during periods of low endogenous estradiol (12, 21), and blocking oestrogen receptors via an oestrogen receptor antagonist impairs extinction recall in female rats (15), suggesting that fear extinction may be dependent, at least in part, on estradiol in females.

Notably, however, reproductive experience (encompassing pregnancy, parturition, lactation, and maternal behaviour) leads to long-lasting changes in the nature of estradiol fluctuations across the estrous/menstrual cycle. For example, young adult primiparous rats (one reproductive experience) have lower levels of circulating estradiol compared to nulliparous rats (no reproductive experience) across the estrous cycle (17, 22) and parous women have lower estradiol levels than nulliparous women across the menstrual cycle (23). Moreover, we have recently shown that reproductive experience alters the modulating effect of estradiol on fear extinction. In contrast to nulliparous female rats who exhibit poorer extinction recall when extinguished during the metestrus (i.e., low endogenous estradiol) compared to the proestrus (i.e., high endogenous estradiol) phase of the estrous cycle, primiparous female rats show comparable extinction recall irrespective of the estrous phase in which they were extinguished, even when extinguished 3 months post-weaning (17, 24). In addition, systemic administration of estradiol enhances fear extinction in nulliparous female rats but has no effect in primiparous females (25). Similar findings have also been documented in humans, in whom there was a negative correlation between endogenous estradiol levels and fear responses during extinction recall in non-mothers, whereas no such correlation was found in mothers (17). Together, these results suggest that fear extinction may switch from being modulated by estradiol to being independent of estradiol following reproductive experience. Reproductive experience also appears to alter the neurobiological mechanisms underlying fear extinction. For example, unlike nulliparous rats, primiparous rats show fear extinction that is resistant to relapse phenomena (17), independent of the activation of N-methyl-D-aspartate receptors (26), and resistant to the augmenting effects of the pharmacological adjunct D-cycloserine (25). Together, this growing body of work demonstrates that the hormonal, behavioural, and neurobiological features of fear extinction may be altered as a result of reproductive experience.

The factors that contribute to the observed changes in fear extinction following reproductive experience are yet to be investigated. It is possible that these changes are due to pregnancy alone, maternal experience during the postpartum period alone (i.e., interactions with offspring from parturition until weaning), or a combination of the two. Previous studies have found mixed evidence for the role of pregnancy vs. maternal experience on other fear-related behaviours, such as anxiety-like behaviour. Similar to fear extinction, reproductive experience appears to alter anxiety-like behaviour in a long-lasting manner. For instance, some (but not all) studies have found that primiparous rats exhibit reduced anxiety-like behaviour compared to nulliparous rats, even up to 22 months of age [(27–29); but see (30, 31)]. There is some evidence to suggest that maternal experience may be important for such changes as the temporary separation from pups prior to testing increased anxiety-like behaviour in lactating primiparous rats compared to lactating primiparous rats that were not separated from pups (32, 33), and pup fostering reduced anxiety-like behaviour in nulliparous female rats (34). Contrastingly, the permanent removal of pups immediately after parturition had no effect on anxiety-like behaviour in primiparous rats tested 4 weeks after birth, suggesting that the effects of maternal experience on anxiety-like behaviour may not be long-lasting (30).

Studies investigating naturally-occurring variation in mother-pup interactions during the postpartum period have similarly produced inconsistent results. In rodents, there are natural variations in maternal care behaviours such as licking/grooming, nursing postures (e.g., arched-back nursing), and contact with the nest/pups, which are associated with altered emotionality in the offspring and mother (35, 36). For instance, studies have found that lactating dams bred for either low- or high- anxiety-like behaviour differ in maternal behaviours, such that high-anxiety dams spend more time on the nest/pups and arched-back nursing pups than low-anxiety dams (37, 38). In contrast, another study found a negative association between arched-back nursing and anxiety-like behaviour in primiparous rats post-weaning on one task (i.e., the open field task) but no relationship was found on another task (i.e., elevated plus maze) (30). Anxiety-like behaviour in primiparous rats may be influenced by methodological factors such as strain, paradigms used to measure anxiety-like behaviour, or time of testing relative to parturition (i.e., pre- or post-weaning). Combined, these studies indicate that naturally-occurring variation in mother-pup interactions, and thereby naturally-occurring variation in the maternal experience, may have short- and long- term effects on maternal emotionality.

The current study sought to examine whether maternal experience during the postpartum period is necessary for the changes in fear extinction following reproductive experience. In Experiment 1, we investigated whether the dissociable impact of estrous phase on fear extinction following reproductive experience is due to pregnancy alone or a combination of both pregnancy and maternal experience (i.e., exposure to pups). In Experiment 1a, we tested whether we could replicate the finding that fear extinction is estrous-cycle dependent in nulliparous but not primiparous female rats. In Experiment 1b, we tested whether estrous cycle effects on fear extinction would re-emerge in primiparous rats that had their pups permanently removed within 24 h of parturition. If maternal experience is necessary to abolish estrous effects on fear extinction, then primiparous rats who experienced pregnancy, but not pup exposure, should exhibit poorer extinction recall when extinguished during metestrus compared to proestrus. In Experiment 2, we investigated whether naturally-occurring variations in maternal behaviour (i.e., mother-pup interactions) predict future performance in fear extinction and anxiety-like behaviour in primiparous rats. We measured maternal care behaviours toward pups during the first week of lactation, and then primiparous rats were tested for anxiety-like behaviour and fear extinction post-weaning. If natural variation in maternal experience contributes, in part, to variability in future expression of fear extinction and anxiety-like behaviour in primiparous rats, then maternal care behaviours should be significantly correlated with fear extinction outcomes and anxiety-like behaviour.

## Materials and Methods

### Animal Subjects

Experimentally naïve nulliparous and primiparous female Sprague-Dawley rats were obtained from the Animal Resources Centre (ARC), Perth, WA, Australia. Upon arrival, rats were housed in groups of 5 to 8 in plastic boxes (67 cm long × 30 cm wide × 22 cm high) filled with corncob bedding and covered with a wire lid. The boxes were kept in a 20–22°C colony room maintained on a 12 h light–dark cycle (lights on at 0700 h), and food and water were available *ad libitum*. Rats continued to be housed under these conditions for an acclimatisation period of ~2 weeks prior to the commencement of any procedures. Rats were age-matched (24–28 weeks old) in all experiments. Procedures were approved by the Animal Care and Ethics Committee at UNSW Australia and followed guidelines in *The Australian Code of Practice for the Care and Use of Animals for Scientific Purposes* (8th edition, 2013).

### Estrous Cycle

Vaginal smears were conducted daily (between 0900 and 1100 h) to determine estrous phase as previously described (11). A regular 4–5 day estrous cycle consists of four phases: metestrus, diestrus, proestrus, and estrus. Vaginal smears began on the first day of handling and ended on the final day of behavioural testing to ensure regular cycling across experiments. Vaginal smears and handling were conducted at the same time for all groups in each experiment. Handling began 1 month after weaning in primiparous rats, once lactation had ceased and estrous cycling had recommenced (17).

### Apparatus

#### Conditioning and Extinction Chambers

Two sets of experimental chambers served as distinct contexts for conditioning (Context A) and extinction procedures (Context B). These chambers differed by visual and tactile features, as previously described (11). The CS was a 62 dB white noise delivered through a speaker on the wall of each chamber, and the US was a 0.4 mA, 1.0 s footshock delivered through the floor. A computer running Med Associates Med-PC IV controlled presentations of both the CS and the US.

#### Light Dark Box

The light-dark box (LDB) was made of two Perspex compartments (each compartment 18 cm wide × 18.5 cm long × 20 cm high). The dark compartment had black opaque walls and a black lid (1 lux), while the light compartment had white opaque walls and was illuminated by a ceiling light through a clear lid (85 lux). The lighting conditions were chosen based on pilot studies in our lab showing variance in anxiety-like behaviour not at floor or ceiling. There was a small opening (7.5 cm × 7.5 cm) between the two compartments that could be blocked with a sliding metal door. A video camera positioned above the apparatus was used to record animal behaviour for later scoring.

#### Elevated Plus Maze

The elevated plus maze (EPM) consisted of two open arms and two closed arms that met at the centre to form a cross, elevated 53 cm off the floor (arms were 113 cm long × 10.5 cm wide; walls on closed arms were 40 cm high). The EPM was illuminated by a ceiling light and two lights positioned on the floor (300 lux). The lighting conditions were chosen based on pilot studies in our lab showing variance in anxiety-like behaviour not at floor or ceiling. A video camera positioned above the apparatus was used to record animal behaviour for later scoring.

### Experiment 1 Procedure and Scoring

#### Breeding

**Figure 1:**
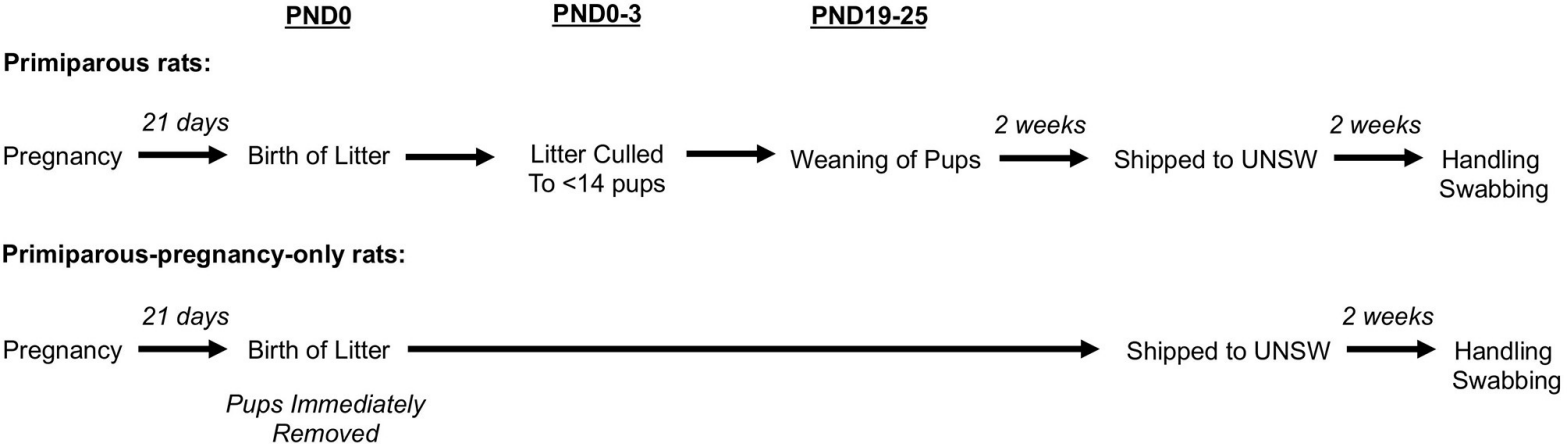
Experiment 1a and 1b timeline of breeding in primiparous rats and primiparous-pregnancy-only rats.

Breeding occurred at the ARC, prior to the arrival at UNSW. Nulliparous female Sprague-Dawley rats were housed with a single sexually experienced male rat for up to 5 days until a vaginal plug had been observed. Pregnant females were housed in groups of 10. After 2.5 weeks, pregnant females were individually housed in maternity boxes until the birth of their litter (maximum of 3 days), which was designated as postnatal day (PND) 0. Rats gave birth to litters ranging in size from 10 to 17 pups. On PND0-3, litters were culled to no more than 14 pups. Primiparous rats were housed with their litters until weaning at approximately PND19-25. Following weaning, primiparous rats were housed in groups of two until they were shipped to UNSW. Nulliparous rats remained as virgins and were housed in groups of up to 15 until they were shipped to UNSW. In Experiment 1a, subjects were nulliparous rats with no reproductive experience or primiparous rats with one prior reproductive experience. In Experiment 1b, subjects were primiparous rats who remained with their pups until weaning (primiparous), or primiparous rats who had their pups permanently removed within 24 h of parturition (primiparous-pregnancy-only). Behavioural testing began ~1 month postweaning in primiparous rats (or 2 months after parturition in primiparous-pregnancy-only rats). For an overview of the timeline of breeding (see Figure 1).

#### Handling and Context Pre-exposure

Experiment 1a and Experiment 1b were conducted at different times. In both experiments, rats were handled for 5 min each day for three consecutive days; after handling on the final 2 days, rats were individually placed in Context A for 10 min to habituate to the conditioning chamber. All behavioural procedures and testing occurred between 1000 and 1400 h.

#### Fear Conditioning

Fear conditioning was identical in Experiments 1a and 1b. Rats were placed in Context A and after a 2 min adaptation period the CS was presented for 10 s and co-terminated with the shock US. Rats received three CS-US pairings (intertrial interval 85–135 s, with an average of 110 s). Rats underwent fear conditioning in either estrus (for groups extinguished during metestrus) or diestrus (for groups extinguished during proestrus). Note that rats in Experiments 1a and 1b were also tested on measures of anxiety-like behaviour (i.e., open field test, LDB, and EPM) 7 days prior to undergoing fear conditioning; those results will be reported in a different paper (Pestana & Graham, in prep.).

#### Extinction Training

Extinction training in Experiments 1a and 1b was identical. Rats underwent extinction training 24 h after conditioning in Context B. After a 2 min adaptation period, rats received 30 × 10 s CS presentations, with an intertrial interval of 10 s. Rats underwent extinction training in either metestrus or proestrus. This design meant that metestrus and proestrus groups were in different estrous phases during fear conditioning and extinction recall but was advantageous in that it allowed for the assessment of estrous phase on fear extinction, whilst keeping the length of time between experimental phases consistent. Importantly, previous research in nulliparous rats has shown that differences between metestrus and proestrus groups (based on estrous phase during extinction training) were evident at extinction recall irrespective of whether estrous phase during fear conditioning and extinction recall was held constant or free to vary (11, 15). There were four groups in Experiment 1a based on the estrous phase during extinction training: Nulliparous-Metestrus (*n* = 11), Nulliparous-Proestrus (*n* = 9), Primiparous-Metestrus (*n* = 12), and Primiparous-Proestrus (*n* = 13). Likewise, there were four groups in Experiment 1b: Primiparous-Metestrus (*n* = 12), Primiparous-Proestrus (*n* = 11), Primiparous-Pregnancy-only-Metestrus (*n* = 12), and Primiparous-Pregnancy-only-Proestrus (*n* = 12).

#### Extinction Recall

Rats were returned to Context B 24 h after extinction training. Following a 1-min adaptation period, rats received a single 2 min CS presentation. Rats were tested for extinction recall in either diestrus (for groups extinguished during metestrus) or estrus (for groups extinguished during proestrus).

#### Scoring

Conditioned fear was measured using freezing, defined by the absence of movement except those related to respiration (39). Rats were scored as “freezing” or “not freezing” every 3 s using a time-sampling procedure. A percentage score was calculated for each animal to determine the proportion of total observations spent freezing.

#### Statistical Analyses

Two-way ANOVAs with the between-subjects factors of reproductive status and estrous phase in Experiment 1a, and pup exposure and estrous phase in Experiment 1b, were used to assess group differences in pre-CS freezing prior to fear conditioning, extinction training, and extinction recall, as well as CS-elicited freezing during extinction recall. Two-way ANOVAs with repeated measures were used to assess group differences in CS-elicited freezing during fear conditioning and extinction training, with the Greenhouse–Geisser correction used if the assumption of sphericity was violated (as indicated by Mauchly's Test of Sphericity). Planned independent samples *t-*tests with Bonferroni corrections for multiple comparisons (*p* = 0.025) were used to assess estrous cycle effects on fear extinction in nulliparous, primiparous, and primiparous-pregnancy-only groups separately. In particular, CS-elicited freezing during extinction recall was compared between rats extinguished during metestrus and proestrus within each group. In Experiment 1a, two nulliparous rats in metestrus were excluded because they failed to learn the fear association (i.e., freezing was <30% in Block 1 of extinction training), and one nulliparous rat in proestrus was excluded on the basis that it was a statistical outlier at extinction recall (i.e., >3 STDEVs away from the mean).

### Experiment 2 Procedure and Scoring

#### Breeding

Breeding occurred at the School of Psychology, UNSW. Nulliparous female Sprague-Dawley rats obtained from ARC were housed in groups of 2–3 in standard cages with a single sexually-experienced male rat. After 3 weeks, pregnant rats were individually housed in “maternity” boxes (24.5 cm long × 37 cm wide × 27 cm high) until the birth of their litter on PND0. Rats gave birth to litters ranging in size from 8 to 17 pups. On PND0, rats and their litter were transferred to an observation chamber to record maternal behaviour. Mothers and their litter remained in the observation chamber and were not disturbed until PND6 when they were transferred back to the “maternity” boxes in the colony room. On PND7, litters were culled to 8 pups with ~ 5 male and 3 female pups. These pups were later used in subsequent unrelated experiments. Primiparous rats were housed with their litters until weaning at PND28. After weaning, primiparous rats (*n* = 31) were housed in groups of 2–4 in standard cages.

#### Maternal Care Observation

Observation chambers (30 cm wide × 30 cm long × 23 cm high) were made of clear Perspex walls and filled with sawdust bedding and shredded paper for nesting, and were housed in wood cabinets to minimise noise disruption with the front wall removed to allow light in. Infrared cameras positioned above and at the back of the chamber recorded mother-pup interactions for later scoring. Similar to Kan and Richardson (40), maternal behaviour was observed over five 60-min observation periods from PND1-6 daily. Three periods of observation occurred during the light phase (0900, 1300, and 1700) and two periods occurred during the dark phase (0600 and 2100) of the light/dark cycle. Within each observation period, each mother was scored every 5 min (12 observations/period × 5 periods per day = 60 observations/mother/day) for the following behaviours: nursing in an arched-back posture (arched-back nursing), nursing whilst laying on pups (blanket nursing), nursing whilst engaged in other activities (e.g., eating/drinking) or whilst laying on her back or side (passive nursing), licking and grooming of pups (licking/grooming), and no physical contact with pups (no contact). All nursing postures and licking/grooming are reflective of maternal care as they involve mother-pup interactions whereas no contact is a form of non-maternal care behaviour (41). Although relatively rare, the maternal care behaviours were not always mutually exclusive in that the dam could be simultaneously arched-back nursing and licking/grooming (35). Observations were converted into a percentage score to indicate the proportion of time a mother was engaged in each behaviour. Observations were scored by JP (~ 30%), TM (~30%) and SH-J (~40%). Observers were trained to a high level of agreement prior to scoring. A random sample of ~30% of the data was cross-scored, and the inter-observer reliability for arched-back nursing was *r*_(7)_ = 0.86 (*p* = 0.003) and for licking/grooming was *r*_(7)_ = 0.92 (*p* < 0.001).

#### Pup Retrieval Test

**Figure 2:**
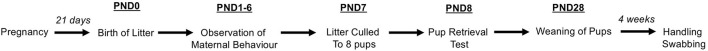
Experimental 2 timeline of breeding and observations of maternal care behaviour in primiparous rats.

The pup retrieval test occurred between 0900 h and 1300 h. The pup retrieval test was adapted from previous research (36, 40). On PND8, pups were removed from the home cage and placed in a holding box for 5 min, while the mother remained in the home cage. Pups were then placed back into the home cage on the opposite side of the nest. The time taken to retrieve the entire litter back to the nest was recorded by an experimenter standing nearby. Pup retrieval is a challenging test of maternal care as it involves disruption to the nest by briefly removing pups which activates the dam to retrieve her pups. A shorter latency to retrieve the litter is reflective of maternal responsivity, which is an important feature of maternal care (42). The pup retrieval test was scored by TM (~90%) and SH-J (~10%). A random sample of ~25% of the data was cross-scored, and the inter-observer reliability was *r*_(6)_ = 0.99 (*p* < 0.001). For an overview of the timeline of maternal behaviour observations (see Figure 2).

#### Handling and Context Pre-exposure

Handling and context pre-exposure was identical to Experiment 1.

#### Anxiety-Like Behaviour

Each rat was individually tested for anxiety-like behaviour in a separate testing room while the remaining rats stayed in their home cage, located in the corridor of the laboratory. Although this testing procedure could introduce variance in anxiety-like behaviour due to emotional contagion, we decided to do this to avoid potential effects of social isolation during periods of testing. Rats were tested on the LDB between 1200 and 1600 h. At test, the animal was confined in the dark compartment for 1-min by blocking the opening with a sliding metal door. The experimenter then removed the door which allowed access to the light compartment for 5-min. Anxiety-like behaviour was indicated by a longer latency to enter the light compartment, a lower number of entries into the light compartment, and a shorter time spent in the light compartment (43). An entry was considered when all four paws of the animal had entered the light compartment. One h after the LDB, rats were tested on the EPM. The animal was placed in the centre of the maze facing an open arm and was observed for 5 min. A lower number of entries into the open arms and a shorter time spent in open arms are indicative of anxiety-like behaviour, whereas the number of entries into the closed arms reflects differences in locomotor activity (44, 45). An entry was considered when all four paws of the animal had crossed into an arm. The LDB and EPM were cleaned with 70% ethanol between tests. After the EPM, rats remained in their home cage for a 7-day rest period prior to undergoing fear conditioning.

#### Fear Conditioning

Conditioning was identical to Experiment 1.

#### Extinction Training

Extinction training was identical to Experiment 1, with the only exception being that rats underwent extinction training during randomised phases of the estrous cycle. This was because the question of interest was to investigate the predictors of fear extinction, which is independent of estrous cycle in primiparous rats (17, 24, 46), and primiparous rats without pup exposure (as demonstrated by Experiment 1b). Based on estrous phase during fear extinction 8 rats were in proestrus, 9 rats were in diestrus, 6 rats were in metestrus, and 8 rats were in estrus.

#### Extinction Recall

Extinction recall was identical to Experiment 1.

#### Statistical Analyses

Pearson's bivariate correlations were used to assess the relationship between maternal behaviour and the following fear indices: anxiety-like behaviour in the LDB as defined by the latency to enter the light compartment, time spent in the light compartment, and the number of entries in the light compartment; anxiety-like behaviour in the EPM as defined by the number of entries in the open arms and the time spent in the open arms; conditioning recall as defined by CS-elicited freezing on the first five-trial block of extinction training; extinction rate as defined by the first extinction trial in which the subject showed 0% CS-elicited freezing, with rats that did not reach 0% freezing on any extinction trial being assigned the total number of extinction trials; and extinction recall as defined by CS-elicited freezing at extinction recall. Similar to Champagne et al. (35), dams were designated as either high or low ABN/LG mothers based on the mean and standard deviation of the ABN and LG scores for the entire cohort. High ABN/LG mothers were defined as females whose frequency scores for both LG and ABN were >0.5 standard deviations above the mean (*n* = 5) whereas low ABN/LG mothers were defined as females whose frequency scores for both LG and ABN were >0.5 standard deviations below the mean (*n* = 4). *Post-hoc* individual *t-*tests were used to compare the above-mentioned fear indices between high ABN/LG mothers and low ABN/LG mothers. Due to the small n's when combining ABN and LG scores, we also used *post-hoc* individual *t-*tests to compare fear indices between high ABN mothers (*n* = 10) and low ABN mothers (*n* = 10), as well as high LG mothers (*n* = 9) and low LG mothers (*n* = 10) based on the same criteria above. One-way ANOVAs with repeated measures were used to measure CS-elicited freezing across fear conditioning and extinction training, with the Greenhouse–Geisser correction used if the assumption of sphericity was violated (as indicated by Mauchly's Test of Sphericity). An exploratory one-way ANOVA with the factor of estrous phase during extinction training (i.e., metestrus, diestrus, proestrus, or estrus) was used to assess the impact of estrous cycle phase on CS-elicited freezing at extinction recall.

## Results

### Experiment 1a

#### Conditioning

**Figure 3:**
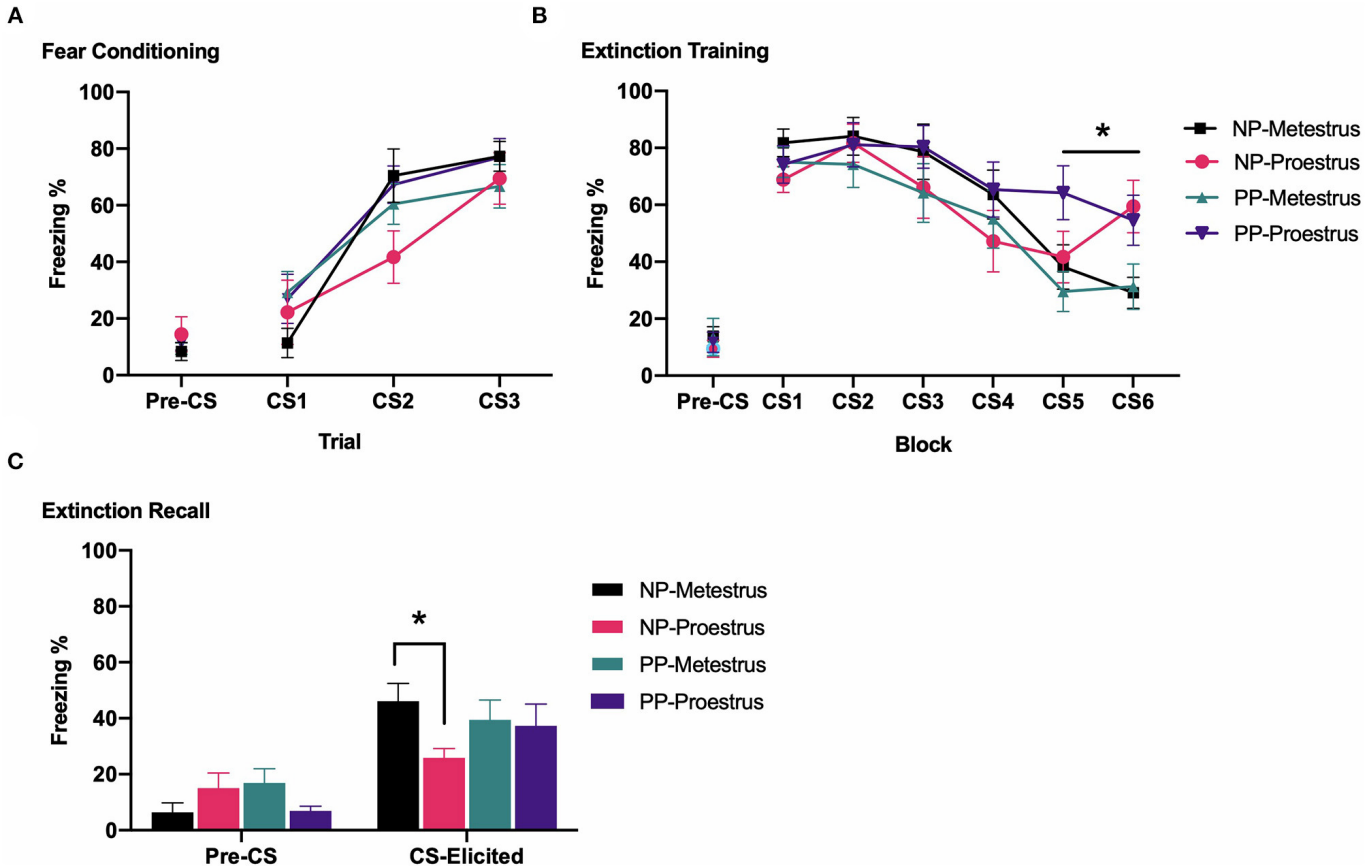
In Experiment 1a, nulliparous and primiparous rats underwent fear conditioning, fear extinction during either metestrus or proestrus, and extinction recall. **(A)** Mean (±SEM) levels of pre-CS and CS-elicited freezing for groups Nulliparous-Metestrus (*n* = 11), Nulliparous-Proestrus (*n* = 9), Primiparous-Metestrus (*n* = 12), and Primiparous-Proestrus (*n* = 13) during fear conditioning. **(B)** Mean (±SEM) pre-CS and CS-elicited freezing for groups during extinction training. The data are presented as 6 blocks of trials, each representing an average of five trials. *Proestrus groups < Metestrus groups during block 5 and 6 of extinction training (*p* < 0.05). **(C)** Mean (±SEM) pre-CS and CS-elicited freezing for groups during extinction recall. *Nulliparous-Proestrus rats < Nulliparous-Metestrus rats (*p* < 0.05). NP, Nulliparous; PP, primiparous.

Figure 3A depicts pre-CS and CS-elicited freezing during fear conditioning. All groups showed comparable pre-CS freezing prior to fear conditioning [largest *F*_(1,41)_ = 1.330, *p* = 0.256]. CS-elicited freezing increased across fear conditioning [*F*_(2,82)_ = 53.725, *p* < 0.001]. There were no main effects of estrous phase or reproductive status, and no interaction between factors [largest *F*_(1,41)_ = 1.605, *p* = 0.212].

#### Extinction

Figure 3B depicts pre-CS and CS-elicited freezing during extinction training. All groups showed comparable pre-CS freezing prior to fear extinction (Fs < 1). Freezing decreased across extinction training [F_(5,205)_ = 27.102, *p* < 0.001]. There were no main effects of estrous phase or reproductive status, and no significant estrous phase × reproductive status interaction, but there was a significant estrous phase × extinction block interaction [*F*_(5,205)_ = 4.552, *p* = 0.002]. This was due to proestrus groups displaying higher CS-elicited freezing than metestrus groups on block 5 [F_(1,41)_ = 4.960, *p* = 0.031] and block 6 of extinction training [F_(1,41)_ = 10.944, *p* = 0.002].

#### Extinction Recall

Figure 3C depicts pre-CS and CS-elicited freezing during extinction recall. All groups showed comparable pre-CS freezing prior to extinction recall [largest *F*_(1,41)_ = 2.921, *p* = 0.095]. During extinction recall, there were no main effects of estrous phase or reproductive status, and no significant estrous phase × reproductive status interaction [largest *F*_(1,41)_ = 2.651, *p* = 0.111]. Planned independent samples *t-*tests revealed that, replicating past research, nulliparous-proestrus rats had significantly lower CS-elicited freezing compared to nulliparous-metestrus rats [*t*_(18)_ = 2.839, *p* = 0.013], whereas primiparous-proestrus and primiparous-metestrus rats did not differ [*t*_(23)_ = 0.195, *p* = 0.847].

### Experiment 1b

#### Conditioning

**Figure 4:**
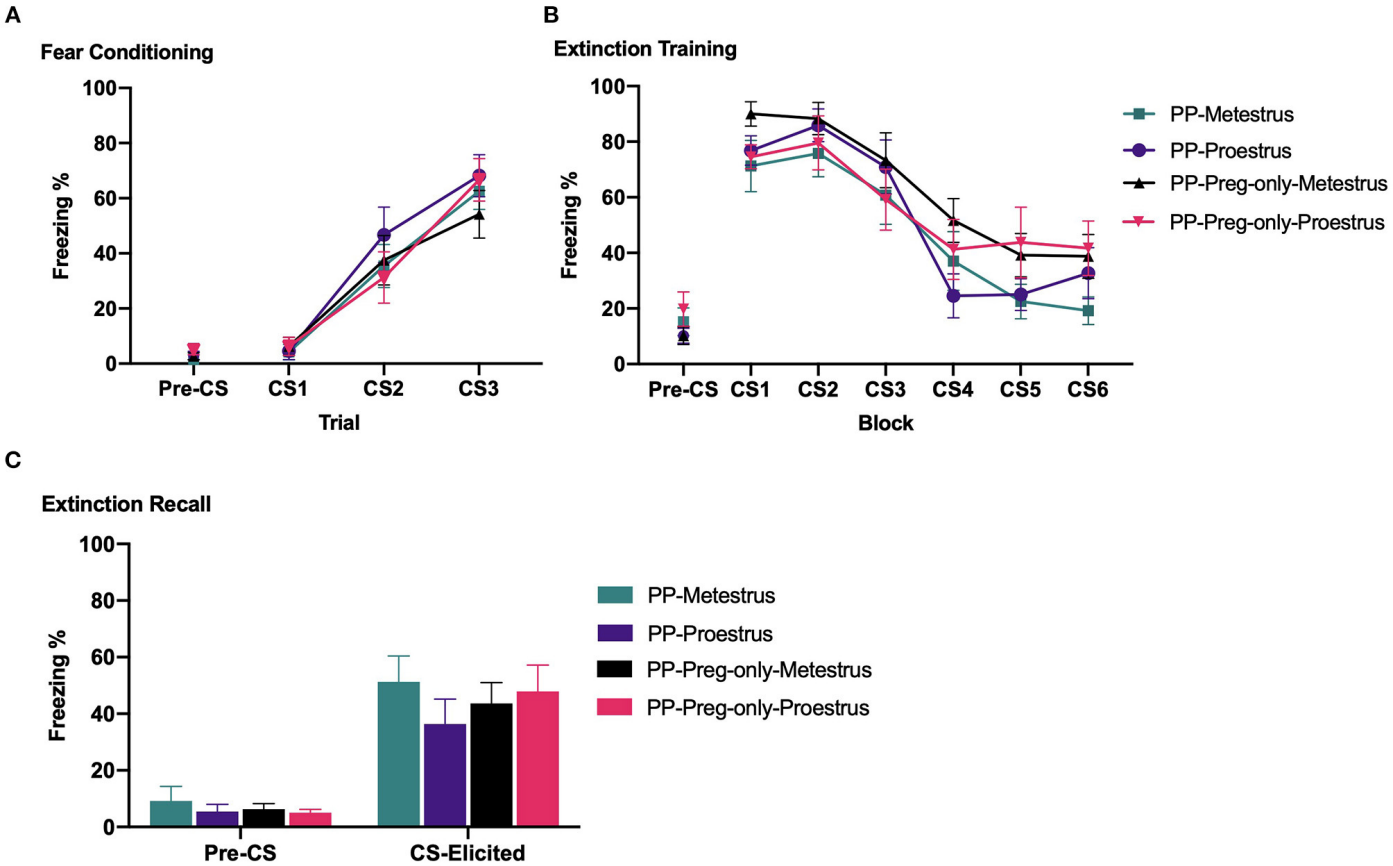
In Experiment 1b, primiparous and primiparous-pregnancy-only rats underwent fear conditioning, fear extinction during either metestrus or proestrus, and extinction recall. **(A)** Mean (±SEM) levels of pre-CS and CS-elicited freezing for groups Primiparous-Metestrus (*n* = 12), Primiparous-Proestrus (*n* = 11), Primiparous-Pregnancy-only-Metestrus (*n* = 12), and Primiparous-Pregnancy-only-Proestrus (*n* = 12) during fear conditioning. **(B)** Mean (±SEM) pre-CS and CS-elicited freezing for groups during extinction training. The data are presented as 6 blocks of trials, each representing an average of five trials. **(C)** Mean (±SEM) pre-CS and CS-elicited freezing for groups during extinction recall. PP, primiparous; Preg, pregnancy.

Figure 4A depicts pre-CS and CS-elicited freezing during fear conditioning. All groups showed comparable pre-CS freezing prior to fear conditioning [largest *F*_(1,43)_ = 1.991, *p* = 0.165]. CS-elicited freezing increased across fear conditioning [*F*_(2,86)_ = 75.399, *p* < 0.001]. There were no main effects of estrous phase or pup exposure, and no estrous phase × pup exposure interaction (Fs<1).

#### Extinction

Figure 4B depicts pre-CS and CS-elicited freezing during extinction training. All groups showed comparable pre-CS freezing prior to extinction training [largest *F*_(1,43)_ = 2.573, *p* = 0.116]. CS-elicited freezing decreased across extinction training [*F*_(5,215)_ = 48.595, *p* < 0.001]. There were no main effects of estrous phase or pup exposure, and no interaction between factors [largest *F*_(1,43)_ = 2.730, *p* = 0.106].

#### Extinction Recall

Figure 4C depicts pre-CS and CS-elicited freezing during extinction recall. All groups showed comparable pre-CS freezing prior to extinction recall (Fs<1). During extinction recall, there were no main effects of estrous phase or pup exposure, and no estrous phase × pup exposure interaction [largest *F*_(1,43)_ = 1.315, *p* = 0.258]. Planned independent samples *t-*tests revealed that, replicating Experiment 1a, primiparous-metestrus and primiparous-proestrus rats showed comparable CS-elicited freezing at extinction recall [*t*_(21)_ = 1.179, *p* = 0.252]. Likewise, primiparous-pregnancy-only-metestrus and primiparous-pregnancy-only-proestrus rats did not differ [*t*_(22)_ = 0.393, *p* = 0.698].

### Experiment 2

#### Descriptive Statistics

**Figure 5:**
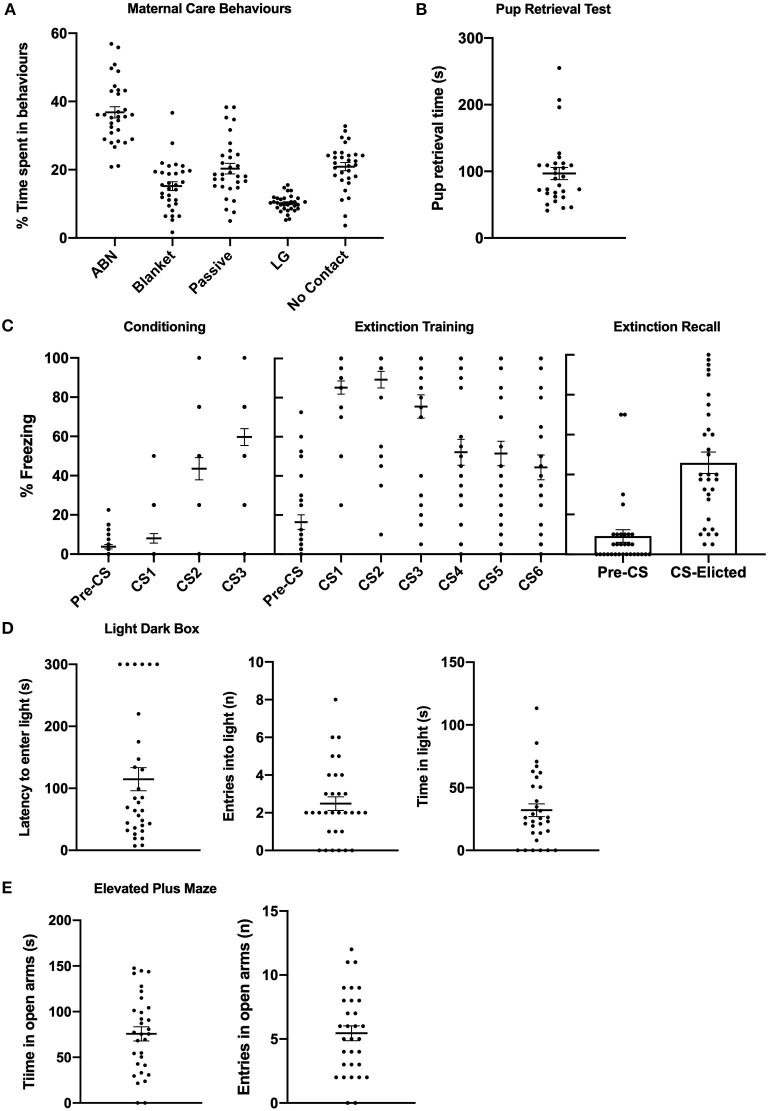
Mean (±SEM) and individual data from primiparous rats (*n* = 31) in Experiment 2. **(A)** % of time spent engaged in various categories of maternal care behaviours. **(B)** Time taken to retrieve last pup in the pup retrieval test. **(C)** Levels of pre- and CS-elicited freezing during conditioning, extinction training, and extinction recall. **(D)** Latency to enter the light compartment, number of entries in the light compartment, and time spent in the light compartment of the light dark box. **(E)** Time spent in the open arms and number of entries in the open arms of the elevated plus maze. ABN, arched-backed nursing; LG, licking/grooming.

Figures 5A,B depicts means and individual data for maternal care behaviours during the observation period and time taken to retrieve the last pup in the pup retrieval test. Means for various categories of maternal behaviour were consistent with previous literature (39, 45). Figure 5C depicts means and individual data for pre-CS and CS-elicited during fear conditioning, fear extinction, and extinction recall. Figures 5D,E depicts means and individual data for anxiety-like behaviour in the LDB and EPM.

#### Conditioning and Extinction

CS-elicited freezing significantly increased across fear conditioning [*F*_(2,60)_ = 44.298, *p* < 0.001], and significantly decreased across extinction training [*F*_(5,150)_ = 20.265, *p* < 0.001]. The mean (±SEM) extinction rate was 19.26 (1.43). Mean levels of CS-elicited freezing during extinction recall were consistent with primiparous rats in Experiments 1a and 1b, and were also independent of estrous phase [*F*_(3,30)_ = 0.891, *p* = 0.458].

#### Relationships With Maternal Care Behaviour

Table 1 reports correlations between maternal behaviours and fear indices. There were no significant correlations between maternal care behaviours and anxiety-like behaviour on either the LDB or EPM (smallest *p* = 0.160). Likewise, there were no significant correlations between maternal care behaviours and conditioning recall, extinction rate, or extinction recall (smallest *p* = 0.098). There were also no significant correlations between litter size (number of pups birthed) and any fear measure (smallest *p* = 0.406). Table 2 reports means for fear measures in groups that were designated based on ABN and LG scores. *Post-hoc* individual *t*-tests revealed that there were no significant differences between high ABN/LG mothers and low ABN/LG mothers on any fear measure [largest *t*_(7)_ =1.228, *p* = 0.259]. Likewise, there was no significant differences on any fear measure between high ABN and low ABN mothers [largest *t*_(18)_ = 0.621 *p* = 0.542], or high LG and low LG mothers [largest *t*_(17)_ = 1.562, *p* = 0.141].

**Table 1:** Correlations between maternal behaviour and fear indices in primiparous rats (*n* = 31) in Experiment 2.

	**1**.	**2**.	**3**.	**4**.	**5**.	**6**.	**7**.
Arched-back nursing	**–**						
Blanket nursing	−0.046	**–**					
Passive nursing	−0.495^**^	−0.602^**^	**–**				
Licking/grooming	0.281	−0.067	−0.044	**–**			
No contact	−0.620^**^	−0.152	0.072	−0.191	**–**		
Pup retrieval	−0.256	0.355	−0.125	−0.104	0.262	**–**	
Litter size	0.067	−0.294	0.347	0.190	−0.316	−0.438^*^	**–**
LDB latency in light	−0.124	0.203	−0.062	0.038	0.046	0.072	−0.006
LDB time in light	0.07	−0.21	0.058	−0.152	0.004	−0.039	0.023
LDB entries in light	0.167	−0.214	−0.005	−0.065	−0.023	−0.085	0.017
EPM time in open	0.149	−0.013	−0.025	0.093	−0.333	−0.268	0.124
EPM entries in open	0.116	−0.061	−0.014	0.039	−0.321	−0.264	0.155
Conditioning recall	−0.051	−0.133	0.048	0.180	0.254	0.177	0.135
Extinction rate	−0.196	0.028	−0.061	−0.055	0.231	0.313	0.094
Extinction recall	0.042	0.075	−0.112	0.183	0.052	−0.052	0.021

*LDB, light-dark box; EPM, elevated plus maze*.

* *p < 0.05,*

** *p < 0.001*.

**Table 2:** In Experiment 2, dams were designated as a high ABN/LG mother if their frequency score for both LG and ABN were >0.5 standard deviations above the mean, whereas dams were designated as a low ABN/LG mother if their frequency score for both LG and ABN were >0.5 standard deviations below the mean.

	**Groups**					
	**High ABN/LG**	**Low ABN/LG**	**High ABN**	**Low ABN**	**High LG**	**Low LG**
LDB latency in light	104.20 (49.65)	70.25 (28.04)	79.40 (25.57)	82.20 (27.45)	111.33 (37.57)	91.00 (27.15)
LDB time in light	26.19 (11.05)	41.03 (11.73)	37.98 (10.93)	38.52 (6.83)	24.20 (6.75)	45.71 (11.99)
LDB entries in light	2.60 (0.98)	3.00 (0.91)	3.10 (0.74)	2.70 (0.51)	2.33 (0.62)	3.30 (0.80)
EPM time in open	80.64 (18.57)	62.48 (25.55)	85.17 (11.46)	75.89 (12.74)	72.82 (15.33)	79.68 (15.46)
EPM entries in open	6.20 (1.53)	5.25 (2.56)	6.10 (0.82)	5.70 (1.20)	4.89 (1.11)	5.90 (1.13)
Conditioning recall	97.00 (2.00)	92.50 (5.95)	87.00 (7.31)	81.00 (6.32)	93.33 (2.04)	80.50 (7.97)
Extinction rate	18.00 (2.53)	22.75 (2.95)	17.20 (2.29)	19.00 (2.27)	19.56 (2.62)	20.00 (3.14)
Extinction recall	46.50 (12.16)	36.25 (14.23)	42.25 (7.64)	37.50 (11.80)	54.44 (8.74)	40.00 (8.01)

*Based on the same criteria, dams were designated as a low ABN mother or a high ABN mother, as well as a low LG mother or high LG mother. Means (±SEM) of fear measures for groups High ABN/LG (n = 5), Low ABN/LG (n = 4), High ABN (n = 10), Low ABN (n = 10), High LG (n = 9), Low LG (n = 10). ABN, Arched-back nursing; LG, Licking/Grooming*.

## Discussion

The results of the current study demonstrate that maternal experience may not be necessary to alter the hormonal features of fear extinction following reproductive experience reported in past work and replicated here. Experiment 1 found that nulliparous female rats had enhanced extinction recall when extinguished during the proestrus compared to the metestrus phase of the estrous cycle, whereas primiparous rats with and without pup exposure showed comparable extinction recall irrespective of estrous phase in which they were extinguished. Primiparous rats with and without pup exposure also showed similar overall extinction recall. In addition, natural variation in maternal experience does not appear to contribute to variability in future expression of fear extinction or anxiety-like behaviour. Experiment 2 found no correlation between maternal care behaviours and fear extinction indices (fear conditioning, extinction rate, or extinction recall) or anxiety-like behaviour on the LDB or EPM in primiparous rats.

The current study replicates previous findings from our lab showing a dissociable impact of estrous phase on fear extinction following reproductive experience, which has now been demonstrated 12 different times (17, 24, 46). The current study expands these findings by showing that fear extinction becomes estrous-cycle independent even in primiparous rats that were permanently separated from their pups within 24 h of birth. This outcome is consistent with our prior observation of estrous-cycle independent fear extinction in primiparous rats that experienced maternal separation (i.e., pups were removed from dams for 3 h daily from PND2-14) during the postpartum period (24). Maternal separation is similar to pup removal as both procedures disrupt the maternal experience by interfering with mother-pup interactions via the removal of pups. Notably, however, maternal separation impaired extinction recall in primiparous rats (24), whereas pup removal in the current study had no overall effect on extinction recall. One possible explanation for these differences is that the repeated brief separation from pups (i.e., 3 h daily from PND2-14) is a more intense stressor compared to the permanent separation from pups, and consequently leads to greater long-term changes in the dam. Consistent with this idea, numerous studies have shown that maternal separation increases anxiety-like behaviour in primiparous rats post-weaning (47–50) whereas permanent pup removal had no such effect (30).

The findings that primiparous rats exhibit estrous-cycle independent fear extinction irrespective of their postpartum experience [i.e., whether they are exposed to maternal separation, Graham (24), or permanent pup removal - Experiment 1b], indicate that pregnancy, not maternal experience, mitigates the influence of estrous cycle on fear extinction. This outcome may account for our previous findings demonstrating that the impact of reproductive experience on the hormonal modulation of fear extinction is similar in both rats and humans, despite large cross-species differences in postpartum experiences (17). For instance, the postpartum experience differs between rats and humans in that women typically give birth to one child, whereas female rats give birth to a litter of pups, often more than 10. In addition, unlike women who typically (but not always) have a support network to help with caregiving behaviours, the female lab rat rears offspring in isolation. The hormonal profile of the postpartum period also differs cross-species [reviewed in (51)]. For example, rats undergo a postpartum estrus, during which previously low estradiol levels sharply rise following parturition (52, 53), whereas estradiol levels remain low following parturition in women (54). In addition, suckling by pups dramatically increases progesterone in parous rats within 3 days after parturition (55–57) whereas progesterone levels remain low in human mothers for months if the infant is breastfeeding (58). Given these cross-species differences, it is unlikely that the postpartum period (and the associated fluctuations in hormones) can account for the alterations in the hormonal features of fear extinction in both parous rats and women.

The current finding that maternal experience is not necessary for reproductive experience to cause estrous-cycle independent extinction suggests that shifts in fear extinction following reproduction are mediated, perhaps entirely, by pregnancy. In contrast to the postpartum period, the hormonal profile of pregnancy is more consistent across species. For example, in both female rats and women, estradiol and progesterone levels increase during gestation and then plummet around the time of parturition [reviewed in (59)]. These drastic hormonal fluctuations are unparalleled by any other neuroendocrine event in the female reproductive lifespan. For instance, estradiol levels are significantly higher in pregnant rats during late gestation relative to nulliparous rats (60), and estradiol levels increase up to 50-fold over the maximum menstrual cycle level in women during the third trimester (54). The increased exposure to estradiol across pregnancy may underlie the persistent changes in endogenous estradiol documented in parous rats and women (17, 22, 23). For example, primiparous rats have lower levels of circulating estradiol compared to nulliparous rats during proestrus [(17, 22); but see (61) for opposite findings in middle age], and human mothers have lower estradiol levels than non-mothers across the menstrual cycle (23). Reproductive experience has also been shown to alter the expression of oestrogen receptors in primiparous rats in brain regions such as the medial amygdala (62). It is possible that the persistent changes in circulating estradiol and oestrogen receptor expression causes the role of estradiol in fear extinction to shift following reproductive experience. One possibility is that estrous effects on fear extinction become harder to detect in primiparous rats given that the natural fluctuations in estradiol become less pronounced following reproduction. To determine the precise role of estradiol fluctuations across pregnancy in mitigating estrous effects on fear extinction, future studies could assess the impact of continuous exposure to high pregnancy-like levels of estradiol in nulliparous female rats (63, 64). If increased exposure to estradiol is necessary and sufficient to cause reproductive-induced shifts in extinction, then nulliparous rats exposed to this procedure should exhibit fear extinction that bears the characteristics of primiparous rats.

The finding that maternal experience was not necessary for the transition to estrous-cycle independent extinction in primiparous rats is conceptually similar to the findings of Experiment 2, which indicated that individual variation in maternal experience had no appreciable relationship with subsequent fear extinction, or anxiety-like behaviour. Likewise, Experiment 1b indicated that fear extinction did not differ between primiparous rats that were exposed to pups and primiparous rats that had no pup exposure. Moreover, across all experiments, the standard errors for fear expression during fear conditioning and extinction were similar in primiparous rats with and without pup exposure, suggesting that interacting with offspring does not produce greater variability on these outcomes. Combined, this reinforces the interpretation that maternal experience has little bearing on subsequent fear extinction. These findings are broadly consistent with the results of studies showing a lack of relationship between mother-pup interactions and future performance on memory- and fear-related tasks. For instance, primiparous rats show enhanced spatial working and reference memory compared to nulliparous rats up to 18 months post-weaning (28, 31, 65–67), however, this effect does not appear to be driven by maternal experience as there is no correlation between maternal care behaviours and spatial memory in primiparous rats 1 month post-weaning (66). In addition, consistent with the current study, Pawluski et al. (30) found no correlation between maternal care behaviours and anxiety-like behaviour on the EPM in primiparous rats 1 week post-weaning. However, in their study there was a negative correlation between arched-back nursing and the number of peripheral crossings on the open field test, suggesting that maternal care behaviours may be associated with anxiety-like behaviour under some circumstances. For instance, it is possible that a relationship between maternal care behaviours and anxiety-like behaviour is only evident on some measures, but not others, in which case, this suggests that the relationship is not robust. Taken together, these findings suggest that although there are naturally-occurring variations in maternal experience during the postpartum period, this variation does not seem to influence memory- and fear-related behaviours in primiparous rats beyond weaning.

While postpartum experiences may not modulate the long-term changes in fear extinction in parous rats and women examined in our current and previous studies, we cannot rule out whether these experiences have an impact on fear extinction during the postpartum period. The impact of estrous/menstrual phase on fear extinction cannot be examined in postpartum rats or women because estrous/menstrual cyclicity often ceases during lactation (68, 69). However, future studies can investigate whether naturally-occurring variations in maternal experience are associated with fear extinction performance in primiparous rats prior to weaning. Indeed, although studies have revealed that the relationship between maternal care behaviours and anxiety-like behaviour may not be robust in primiparous rats post-weaning, maternal care behaviours do seem to be positively correlated with anxiety-like behaviour in lactating primiparous rats that were bred to exhibit anxiety-like behaviour (37, 38).

In summary, the current findings indicate that pregnancy has long-lasting impacts on the underlying mechanisms of fear extinction in females. Although further research is required to determine the precise components of pregnancy that contribute to the alterations in extinction following reproduction, these results indicate that current treatments for anxiety based on extinction processes may operate differently not only in men and women, but also as a function of women's history of pregnancy. As fear extinction forms the laboratory basis of exposure therapy, these findings emphasise the need to re-evaluate our animal models of anxiety to not only consider females, but also consider reproductive experience. Doing so may increase the translatability of our findings from female animals to women, many of which will experience pregnancy during their lifetime.

## Data Availability Statement

The raw data supporting the conclusions of this article will be made available by the authors, without undue reservation.

## Ethics Statement

The animal study was reviewed and approved by Animal Care and Ethics Committee at UNSW Australia.

## Author Contributions

All experiments were designed, conducted, and analysed by JP. RR, TM, and SH-J designed procedures for observations of maternal behaviour. JP, TM, and SH-J performed breeding and scored observations of maternal behaviour. RR performed breeding and provided maternal care observation chambers. Study design, development, and oversight of all experiments were provided by BG. JP wrote the first draft of the article with subsequent edits contributed by JP, TM, SH-J, RR, and BG. All authors contributed to the article and approved the submitted version.

## Funding

This research was supported by an Australian Research Council Discovery project (DP180101563) to BG, an Australian Research Council Discovery project (DP190102975) to RR, and Australian Research Training Program Scholarships to JP and SH-J.

## Conflict of Interest

The authors declare that the research was conducted in the absence of any commercial or financial relationships that could be construed as a potential conflict of interest.

## Publisher's Note

All claims expressed in this article are solely those of the authors and do not necessarily represent those of their affiliated organizations, or those of the publisher, the editors and the reviewers. Any product that may be evaluated in this article, or claim that may be made by its manufacturer, is not guaranteed or endorsed by the publisher.
